# Hospitals under the National Insurance Act

**Published:** 1912-01-20

**Authors:** T. Basil Rhodes

**Affiliations:** Secretary and House Governor North Staffordshire Infirmary. Hartshill, Stoke-on-Trent.


*To the Editor of* The Hospital.

### HOSPITALS UNDER THE NATIONAL INSURANCE ACT.

*To the Editor of* The Hospital.

Sir,—I very sincerely apologise for troubling you with this letter, but as you have done me the honour of including some of my remarks in the Report of the proceedings of the Conference of Representatives of Hospitals and Members of the British Hospitals Association at the Westminster Palace Hotel on December 1 last, reported in your issue of December 9, I should be greatly obliged if you would allow me to correct the report of those remarks, seeing that the omission of one sentence from my actual speech alters the whole impression of my opinions that I desired to convey.

Your report reads :—

"Dr. T. B. Rhodes (North Staffordshire Infirmary) pointed out that by the resolution they had just passed the meeting committed itself to State aid. This being the case, etc., etc."

As a matter of fact, at the conclusion of the first sentence I added the following words—or as nearly those identical words as I can remember; but certainly words meant to convey the same impression :—

"However that may be, this resolution has been passed and should be loyally accepted, and it would therefore be well to take steps to press it upon the Government."

My opinion is, I realise, of very little importance, but I do not like to leave a published report of my own remarks uncorrected when they convey an absolutely wrong impression of what I feel. Rightly or wrongly, I am firmly convinced that the resolution passed by that meeting undoubtedly asks the Government to repay to hospitals the cost of treating insured persons treated therein.

My contention is that this, if acted upon, would inevitably lead to a demand for payment to be made to those medical men who now act as honoraries to some at least of the hospitals, and so—eventually—to the payment of all, or nearly all, such medical men.

This would cost the hospitals a great deal more than the hospitals would receive from the Government (or from any other source than I can see) as payment for the insured persons treated in hospital.

Now, it seems to be generally expected by hospital managers, whether honorary or paid, that the Insurance Bill will cause a great diminution in their income, and I agree that in most cases this diminution may be expected, though at the same time I believe that certain hospitals who have gained the trust and support of the workpeople in the district concerned will find that their trust in the continued support of these toilers has not been misplaced; but, I repeat, my belief is that the majority of such hospitals as depend largely on private munificence and legacies will suffer a great loss of such income. On the top of this loss will come the payment of medical men for their services.

The logical result of this must, in my opinion, be the necessity for large State aid, and consequently State control, possibly partial at first, but finally complete.

Now for inspection and audit by the State. I am prepared at any moment—as I believe practically every hospital manager, whether honorary or paid, is also prepared—in fact, I personally would welcome such, as a splendid method of bringing up to a good standard of efficiency any hospital that may have fallen below such standard; and it is undeniable that from time to time a hospital here and there is found on inquiry to have fallen into comparatively out-of-date conditions of administration and equipment; but I believe most firmly—though possibly erroneously—that the State control of the general hospitals will not be advantageous

(*a*) to the patients—because, amongst other things, the searching light of publicity does not readily penetrate into a State or municipally managed institution, so that while some are extremely well managed, others are extremely badly managed, and too much is left to the personality of the few people in charge of such institutions;

(*b*) to the medical men—because it will tend to place all professional work on a definite hard and fast scale of payment, and will take away one of the honours of the profession—its voluntary help to those who cannot afford to pay for the necessary treatment required; and

(*c*) to the public—because it will establish the principle of inquiry into income, so that certain shall be eligible on monetary grounds only after an inquiry by an established official, and others will be refused after a similar inquiry.

There are many other reasons that one could advance against the principle of State management of general hospitals—such, for instance, as the difficulty the public who can afford to pay for the best operative work will find in obtaining the same except by paying a sum to a hospital; and all of the disadvantages mentioned above under headings (*a*), (*b*), and (*c*), will, in my opinion, interact on one another. But at least I believe it necessary to draw the attention of hospital managers to the way in which —as I understand it—that resolution which was passed on December 1 is leading them, and in regard to which I myself refrained from voting simply and solely from a desire not to hamper the action of the British Hospitals Association; a desire which I hoped to show by advocating that steps should be taken to press the views of the Conference upon the Government (although, as it seemed, I was practically alone in my opposition to the amendment—if I except perhaps the Rev. H. V. Stuart, chairman of the House Committee of the North Staffordshire Infirmary, who also expressed his uneasiness in regard to the amended resolution, while preferring it to the two originally proposed).

I would not have written at such length, but that I know well you have the interests of the hospitals very much at heart, and would always be willing to put forward both sides of the question at issue, even if you could not personally agree with the views expressed; and if you will permit me to trespass on your valuable space a little further, I will venture to suggest a method which in my opinion would postpone for some time the complete State control of general hospitals.

It is this: (*a*) Whensoever the National Insurance Bill comes into force let the general hospitals continue to take those cases which—on the signed certificate of the medical practitioner concerned—cannot be suitably treated at home; whether as in-patients or as out-patients. (The latter would, I imagine, be only the patients in regard to whom the general practitioner required consultative advice.) In any case, the details of the necessary arrangements under this section would have to be such as were approved by the honorary medical staff of each hospital.

(*b*) At the end of the financial year let each hospital be submitted to an examination by an inspector appointed by the State on the undermentioned points, and be certified as "approved," or not, as the case may be.

(1) Has there been during the past year any expenditure which was markedly needless or avoidable?

(2) Has sufficient effort been made by the management to retain or increase the voluntary subscriptions?

(3) Having regard to the condition of the buildings when the National Insurance Bill came into force, does the condition of those buildings show that suitable efforts have been made to set them in proper condition, or maintain them in such condition?

(4) Is the number of beds available in the hospital adequate for the general requirements of the district served, having regard to the amount of money contributed voluntarily from such district?

If, then, the hospital has been "approved" in regard to the above points, the hospital shall be entitled to receive from the Treasury the exact sum of money by which the expenditure for the year has exceeded the income for that year, the State saying, in effect, You have done a good year's work, and in order that your next year's work may not be hampered by having a debt to pay off, and interest to pay on overdraft, we start you "fair" again.

I would, in conclusion, like to state most emphatically that the opinions expressed in this letter are merely my own personal opinions, and are not to be taken as necessarily in any way representing the views of the committee of the hospital I have the honour to serve, nor have I the most remote desire to be regarded as expressing the opinion of any other qualified medical man than myself; but I feel very strongly that the present moment is one of acute crisis for both the voluntary hospitals and for the medical profession, and that every opinion, however absurd, if honestly held, should at least be submitted, and, if thought to be worthy, should be placed in the melting-pot in order that a correct conclusion should be eventually arrived at.

Again apologising for the length of this letter, and hoping that you will find an early opportunity of publishing the same, I remain, yours faithfully,

Hartshill, Stoke-on-Trent. T. Basil Rhodes,

January 4. Secretary and House Governor

North Staffordshire Infirmary.

[The following are the exact words used by Dr. Rhodes as taken down verbatim by our reporter: "Dr. T. B. Rhodes: By this resolution (we have) practically committed ourselves to State aid. Therefore, let us do something with it. I propose that an Executive Committee be formed by the Council to follow out these arguments and press them upon the Government and the Chancellor of the Exchequer."—Ed. The Hospital.]

